# Sleep related painful erection: an algorithm for evaluation and management

**DOI:** 10.1186/s12610-019-0095-5

**Published:** 2019-12-04

**Authors:** Maher Abdessater, Anthony Kanbar, Ahmed S. Zugail, Abdalla Al hammadi, Bertrand Guillonneau, Sebastien Beley

**Affiliations:** Groupe hospitalier Diaconesses - Croix Saint Simon, Paris- France, 5 quai bucherelle, 95300 Pontoise, France

**Keywords:** Erection douloureuse nocturne, algorithme, évaluation, diagnostic, traitement, Sleep related painful erection, SRPE, Algorithm, Management, Treatment

## Abstract

**Background:**

Sleep related painful erection (SRPE) is a rare parasomnia consisting of nocturnal penile tumescence accompanied by pain that awakens the individual. Normal non-painful erections are experienced when awake. No penile anatomic abnormalities are present. No conclusive randomized clinical trial is present in the literature about the management of this rare condition. The aim of this article is to review the current knowledge about the management of SRPE and to suggest an algorithm to help physicians evaluate and manage SRPE.

**Material and methods:**

A literature review was conducted through PubMed database using the terms: sleep, pain, painful, penile, and erection. The reference lists of the articles were also reviewed. The search returned 23 references that were published between 1987 and 2019. Results were presented in a descriptive manner.

**Results:**

Treatment decision for now is based on reports of the treatment success, the sustainability of remission, the tolerability by the patients and the potential side effects of each medication. From data available in literature, Baclofen is the mostly used medication with a tolerable profile of adverse effects. Phosphodiesterase type 5 inhibitors are considered potential treatments and are already widely used and tolerated for other indications, but so far only 2 successful trials have been reported for SRPE. Cinitapride is very promising, but only one case was studied and no side effects were reported. Clozapine can be very dangerous although highly effective.

**Conclusion:**

Based on the limited number of treatment trials and reported cases, the low level of evidence and the lack of randomized clinical trials, no treatment consensus for SRPE can be reached. We suggested a useful tool for clinicians: an algorithm for the management of SRPE to facilitate their access to the literature without exhaustive return to case reports and series upon each case faced.

## RÉSUMÉ

**Contexte:**

L’érection nocturne douloureuse est. une parasomnie, caractérisée par des épisodes de tumescence douloureuse provenant exclusivement lors du sommeil, avec une anatomie normale et des érections indolores lorsque le patient est. réveillé. La prise en charge de cette maladie est. inconnue vue le petit nombre de cas et le manque d’études randomisées. Nous présentons dans cet article une revue de la littérature concentrée sur les stratégies thérapeutiques décrites. Un algorithme est. suggéré pour faciliter l’évaluation et la prise en charge des patients.

**Matériel et méthodes:**

La revue de la littérature a été réalisée en utilisant la base de donnée PubMed et les termes: « sleep, pain, painful, penile, erection ». Les références des articles ont été revues. En total 23 références ont été utiles pour notre revue. Les résultats ont été présentés d’une manière descriptive.

**Résultats:**

Les stratégies thérapeutiques actuelles se basent sur les essais décrits dans la littérature à travers un nombre limité de patients. La durée de la rémission et les effets secondaires minimes rendent certains traitements supérieurs. Le baclofen est. le plus utilisé grâce à son efficacité et à son faible potentiel d’effets secondaires. Le cinitapride a été très efficace lors d’un essai unique chez un seul patient, sans effets secondaires rapportés. Les inhibiteurs de la phosphodiesterase de type 5, largement utilisés en urologie, ont été trouvés utiles pour le traitement des érections nocturnes douloureuses chez 2 patients. La clozapine qui est. très efficace, présente un profil d’effets secondaires dangereux.

**Conclusion:**

A cause du faible nombre de cas et des essais thérapeutiques rapportés, le meilleur traitement des patients ayant des érections douloureuses nocturnes reste indéterminé. Nous suggérons un algorithme utile aux praticiens pour l’évaluation, le diagnostic et la prise en charge de ces patients.

## Introduction

Sleep related painful erection (SRPE) is a rare parasomnia [1], different from nocturnal penile tumescence (NPT) that occurs normally in healthy men several times during rapid eye movement (REM) sleep. NPT is called SRPE when it is accompanied by pain that awakens the individual, who has normal non-painful erections when awake, and when no penile anatomic abnormalities are present [2]. The American Academy of Sleep Medicine defined the disease as a REM parasomnia in the first edition of the International Classification of Sleep Disorders (ICSD) defined the disease in 1990 [3], but didn’t include it in its future editions [4, 5]. Typically, men go through multiple investigations, treatment trials and failure before diagnosis, because they fear to become impotent. A mean delay of 5 years is reported between symptoms onset and diagnosis [1, 6]. So far, treatment is an expert-based opinion [7]. Different therapeutic strategies have been described through literature from 47 patients’ experiences, reported in case reports and series without any conclusive randomized clinical trial.

In this article, we will show the current knowledge about the management of SRPE, and at the end, we will suggest an algorithm to help physicians evaluate and manage SRPE, without the need to return to an exhaustive literature review upon each case.

## Material and methods

**Fig. 1:**
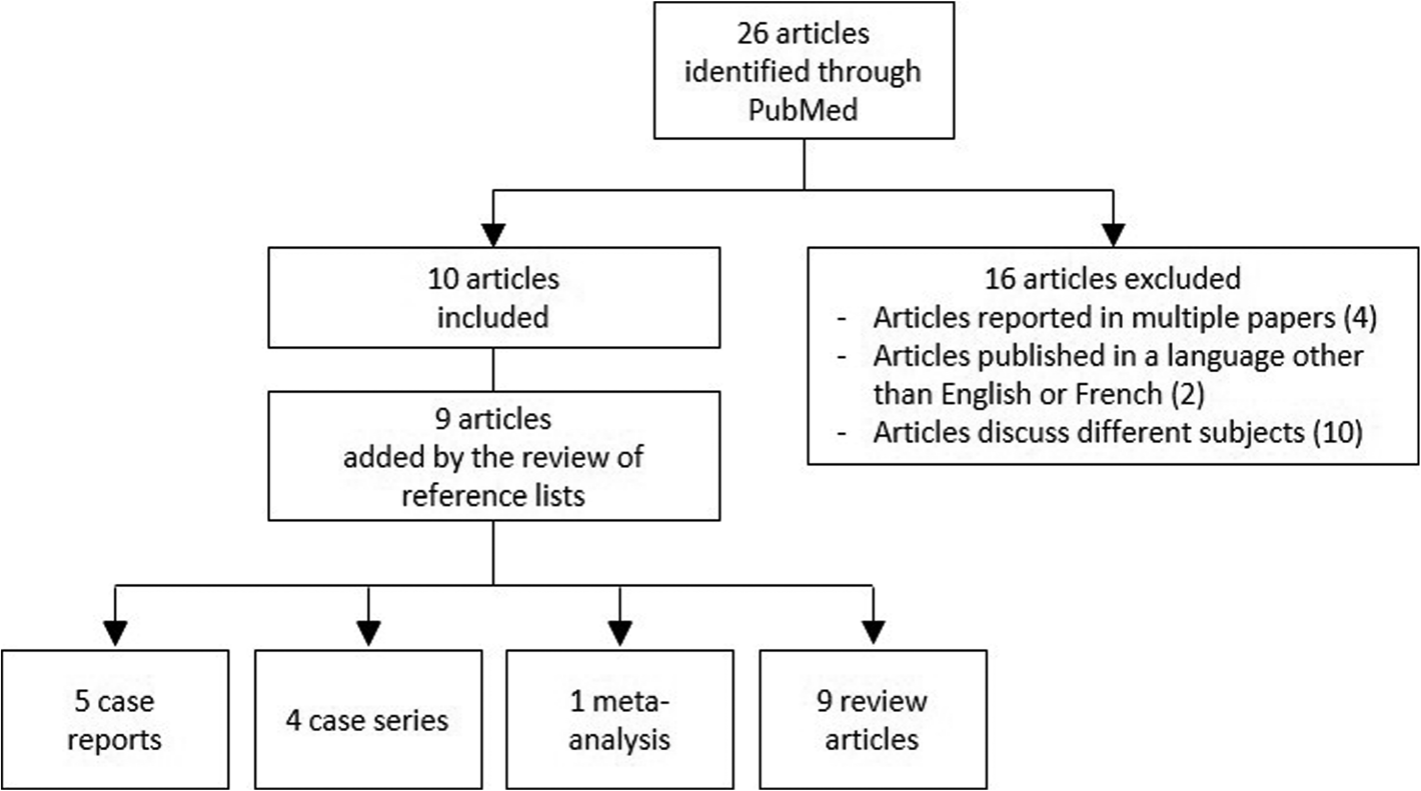
Diagram of literature review process

The literature review was conducted through PubMed database using the terms: sleep, pain, painful, penile, and erection. The reference lists of the articles were also reviewed. The search identified 26 articles published between 1987 and 2019, from which 10 were relevant to our subject. Additional 9 published articles and 4 more references were included after the review of the reference lists of the 10 relevant articles. Excluded articles [8–23] are detailed in Table 1. In total, 5 cases reports, 4 case series, 1 meta-analysis, 9 review articles served for our review (Fig.1). Results were presented in a descriptive manner.

**Table 1:** Excluded articles with detailed reasons of exclusion

Article title	First author	Exclusion reasons
1. REM sleep parasomnias [8].	Schenck CH	Irrelevant subject
2. Cardiac autonomic nervous activity in sleep-related painful erections [9].	Ferini-Strambi L	Information already retrieved from the meta-analysis article written by Vreugdenhil S et al. in 2018
3. Sexual dysfunction in men with multiple sclerosis--a comprehensive pilot-study into etiology [10].	Lottman PE	Irrelevant subject
4. [Painful erections related to sleeping] [11].	Menéndez López V	Article in Spanish
5. Sleep-related painful erection is associated with neurovascular compression of basal forebrain [12].	Szücs A	Information already retrieved from an article written by Karsenty G et al. in 2005
6. Neurological aspects of some sleep disorders [13].	Szúcs A.	Irrelevant subject
7. Radical prostatectomy and quality of life among African Americans [14].	Ukoli FA	Irrelevant subject
8. Subjective symptoms, sleeping problems, and cognitive performance in subjects living near mobile phone base stations [15].	Hutter HP	Irrelevant subject
9. Epithelioid haemangioma: a rare cause of painful erections and sleep deprivation [16].	Lucky MA	Irrelevant subject
10. Prevention of recurrent ischemic priapism with ketoconazole: evolution of a treatment protocol and patient outcomes [17].	Hoeh MP	Irrelevant subject
11. Sleep-related painful erections in an elderly man successfully treated using clonazepam [18].	Kuhadiya ND	We already know from all previous literature before 2104 that SRPE can be treated successfully with clonazepam.
12. [Diagnosis and management of sleep-related painful erections:A report of 9 cases] [19].	Hu HB	Article in Chinese
13. [Considerations on priapism] [20].	Bai WJ	Irrelevant subject
14. Improvement of Erection Related Incision Pain in Circumcision Patients using Interrupted Rapid Eye Movement Sleep: A Randomized Controlled Study [21].	Dai AJ	Irrelevant subject
15. Microcurrent as an adjunct therapy to accelerate chronic wound healing and reduce patient pain [22].	Nair HKR	Irrelevant subject
16. Sleep-related painful erection in a patient with obstructive sleep apnea syndrome [23].	Zhang J	Case report showing association between SRPE and OSAS that was already stated by Ferré et al. in 2012

### Definition of SRPE

SRPE was described for the first time by Karacan in 1971 [24]. It concerns 1% of men presenting with erectile or sexual problems [25]. The mean age at diagnosis is 40 years [6]. Patients experience painful erections several times every night, each time lasting from minutes to hours. Erections respond to non-medical attempts of detumescence using cold showers, walking, abduction and flexion of the hips, cycling, or urinating which was described to be the most effective [1, 6, 7, 26]. Staying in bed usually makes the pain worse [7]. The intensity of erection and internal penile pain increases gradually through the night to reach a maximum in the early morning [2], which is consistent with the typical increase in the duration of REM sleep in the second part of the night [1]. The pain awakens the patient several times every night, leading after a long history of REM sleep deprivation to insomnia, disturbance of daytime performance, irritability and reduced sex drive [1, 2]. Classically, erections during intercourse and masturbation are normal and painless [27], but anxiety and depression are often present due to sexual life performance implications [28].

The ICSD classifies the disease by its severity as mild, moderate and severe according to the frequency of occurrence, as described in Table 2. The condition is considered chronic when the symptoms have been recurring for more than 1 month [27].

**Table 2:** The severity of sleep related painful erections

Severity	Frequency of occurrence
Mild	Once per week
Moderate	Several times per week
Severe	Every night or several times per night

Reference: Ferré A, Vila J, Jurado MJ, Arcalis N, Camps J, Cambrodi R, et al. Sleep-related painful erections associated with obstructive sleep apnea syndrome. Arch Sex Behav. 2012;41:1059–63.

### Pathophysiology

Several theories about the pathophysiology of the SRPE have been proposed. Even though the exact pathology is still not well defined, the advances in the understanding of the pathophysiological mechanisms are guiding the changes in the treatment targets to more effective molecules with fewer adverse effects.

- In the beginning, the successful use of beta blockers in some cases, made investigators think that a reduction in the vagal firing by the heart is behind the increased beta adrenergic activity at the level of the cavernous muscles (CM) [1]. But the fact that the pain is caused by short and incomplete erections, makes a vascular problem less probable and an abnormal neurological signal transmission more probable [2].
- Other authors proposed that a neurological dysfunction is the possible pathophysiology, either locally at the level of the ischiocavernous and bulbocavernous muscles or centrally at the level of the centers of the anterior hypothalamus that control erection [6]. In the former, the resultant increase in the blood pumping into the CM leads to sustained erections [1]. The latter was reported in a case, where the posterior cerebral artery was causing compression on the anterolateral surface of the hypothalamus leading to SRPE [6].
- In one case SRPE was described in a patient surgically treated for a thoracic ependymoma 2 years ago [6].
- The co-existence with obstructive sleep apnea was observed in two cases [27].
- No clear predisposing factors, hereditary patterns or relation to alcohol or food were demonstrated [29, 30].

Studies through the literature have investigated also the relation between REM sleep and SRPE. It was found that most of SRPE, started during REM and continued beyond this phase through awakening, making painful erections and REM sleep two different entities. This finding is illustrated by the use of drugs that typically act on the REM sleep, like the monoamine oxidase inhibitor (MAOI) Brofaromine and the antidepressants. Those drugs suppress the REM sleep but don’t affect the occurrence of SRPE [31, 32]. Similarly, some central nervous system (CNS) lesions described through literature, affected the REM sleep but didn’t affect the erections. These observations confirm that REM sleep and erection are two different mechanisms, and indicate that SRPE is regulated at higher levels than those controlling REM sleep [2, 6].

Currently, the most recently adopted pathophysiological mechanism behind the SRPE is that a hypersensitivity reaction leads to excessive secretion of neurotransmitters, and subsequent excessive cavernous myorelaxation or perineal muscles activity. This, explains the increased duration of erections, but still lacks the explanation of the associated pain [33].

### Evaluation and diagnosis

Polysomnography can make the diagnosis. It shows a fragmented pattern of sleep and confirms insomnia with elevated number of awakening (more than 5 times) from painful erection and a low total sleep time. Normal to low rate of REM sleep and awakening following REM are observed. Erections are often short, incomplete and increased in number (7 to 10). They begin, mostly but not exclusively, during REM sleep and are maintained to an undetermined interval after getting awake [28].

Physical examination is always normal with no nodule, change in elasticity or phimosis. Penile artery ultrasound, hormonal profile and metabolic panel are normal. Neurological exam is mostly normal. Personality or psychiatric disorders are absent except for sleep deprivation related depression. Anxiety is usually present and manifests by psychogenic erectile dysfunction in 23% - in one patient series - where it was related to sleep loss [1, 2].

### Differential diagnosis

Clinical history and physical exam help differentiating the SRPE from other medical conditions that can cause painful erections, such as Peyronie’s disease, priapism and phimosis.

- Peyronie’s disease causes pain with each erection, either awake or asleep, during intercourse and masturbation. It progresses in two phases with an angulation of the penis. On clinical exam and ultrasonography, the plaque and the retraction of the penis are detectable [34].
- Low flow, or ischemic priapism, has very well defined risk factors, and resolution requires always intracavernosal intervention (injections or aspiration) [7, 35].
- Phimosis, Urethritis and metastasis in the corpora cavernosa cause pain not related to erection, with complete different signs and symptoms [36].

### Treatment approaches

So far, there is no one effective definitive treatment for SRPE.

Some authors have observed spontaneous recovery after change in sexual life and affection, and linked recurrence to emotional factors [1].

In 2012, Ferré et al. reported amelioration of symptoms in 2 patients after treating obstructive sleep apnea with continuous positive airway pressure (CPAP). This was achieved in a dependent manner with recurrence of SRPE after withholding CPAP, suggesting a possible effect of blood gases composition on the neurotransmitter activity implicated in the erection process [27].

Five years later, Vreugdenhil et al. reported promising results from pelvic physiotherapy in 3 of 5 patients diagnosed with SRPE [7].

Hypothetical pharmacological approaches are based either on the inhibition of erection or on the improvement of sleep [30]. Multiple physiologic routes of erection lie behind suggested treatment:

- The continuous sympathetic firing that maintains the penis flaccid made authors consider that a decrease in the sympathetic activity releases the parasympathetic cholinergic system. The latter initiates relaxation of the intracavernous muscles using nitric oxide, prostaglandin E1 and calcitonin gene-related peptide. Usefulness of anticholinergics was seen in the reduction of the vasodilation and relaxation of the smooth muscles of the corpora cavernosa [30].
- Noradrenaline that dilates the cavernous bodies through beta receptors and constricts it via alpha receptors, along with the reduction in the vagal activity and tachycardia that were reported during SRPE, made the use of beta stimulation (beta blockers and alpha agonists) as a possible treatment [30, 37].
- The supraspinal induction of erection via dopaminergic release by the medial preoptic area which is controlled at the same level by the serotonin release by the paragigantocellular nucleus which has an inhibitory effect on the erection, elucidated the effect of dopamine antagonists, serotonin agonists and serotonin uptake inhibitors on the inhibition of erection [29].

### Abandoned treatments

The benzodiazepines were previously used because SRPE was associated to anxiety and conjugal problems. Clonazepam, nitrazepam, oxazepam and diazepam had an unstable effect [30].

Opioids (oxycontin, tramadol) and antiepileptics (carbamazepine, pregabalin) were ineffective.

Beta-blocker use was beneficial in some cases but not in others [7]. Additionally, the temporary effect of propranolol (lasting 2 to 12 weeks) and the resulting erectile dysfunction (as an adverse effect) reduced its utility [6, 38].

Antidepressants, known for their suppressive action on the REM sleep, similarly lost their efficacy at 3 months [6]. In fact, fluoxetine and metyrapone showed a dissociative effect, suppressing REM sleep but maintaining NPT [2]. Clomipramine with its anticholinergic blockade of peripheral autonomic nerves reduced REM and NPT3 but subsequently failed early [1]. Amitriptyline efficacy was temporary at high dose, but symptoms recurred after 3 weeks [6]. Trimipramine and biperidene were not successful [2].

Antiandrogens like cyproterone acetate were effective only in one out of ten patients, when used in combination with baclofene. Predictably, it was abandoned early for the adverse effect of the induced hypogonadism state on sexual performance, bone density, and lipid metabolism [7, 30]. The benefit to risk ratio makes antiandrogens use strongly inadvisable.

### Potential treatments

Phosphodiesterase type 5 (PDE5) inhibitors at low doses (sildenafil 25 mg daily or tadalafil 5 mg daily) were studied successfully. PDE5 is responsible for the degradation of cyclic guanosine monophosphate (cGMP). The inhibition of PDE5 increases cGMP levels and consequently prevents detumescence. Paradoxically, daily low doses are known to increase baseline level of cGMP causing restoration of PDE5 level and activity, resulting in decreased erection. This class of medications has been approved for the prevention of priapism. Suggested similarities of the pathophysiological mechanisms between intermittent priapism and SRPE, led to the trial of PDE5 inhibitors in the treatment of the latter. Daily low dose Tadalafil was successful treating in 2 patients (out of 4) suffering from SRPE. Its action regulates the mechanisms of erection at the molecular level. A beneficial effect was observed for long term, up to 5 years. Those medications are generally well tolerated, with minimal side effects limited to headache, dizziness, flushing and nasal congestion or discharge [30, 39].

Cinitapride is an orthopramide with a strong cholinergic and serotoninergic activity but a weak antidopaminergic action, used generally as a gastroprokinetic. The supraspinal mechanisms regulating erection have directed Chiner et al. to treat successfully a 50-years old man with cinitapride by 1 mg daily, during 1 month, followed by 1 mg every 8 h for 5 months. No recurrence was reported after withholding the treatment for 3 months. The patient experienced reduction in the frequency and intensity of SRPE from several times per night every single night to less than 2 nights weekly with only 1 episode of SRPE by night. Sexual function was maintained and no adverse reactions were reported. The main known side effects of this medication are dystonia of the head, neck and tongue, drowsiness and diarrhea [29].

Clozapine which is a neuroleptic, is the initial successful treatment described since years. It was used for its known anticholinergic and sedative effects. Literature reported one successfully treated patient with low dose (25 mg daily) of clozapine with a sustainable response. The patient had normalization of sleep quality and cessation of SRPE with maintenance of normal drive, mood and NPT [2]. Dependence of the drug occurred when the treatment was stopped. The maintenance of NPT eliminated the anticholinergic mechanism, and highlighted the excess sedation to be the principle mechanism of action of the drug. This medication has dangerous side effects; it carries the risk of agranulocytosis, myocarditis and epileptic seizure, making it less encouraging for use [30].

The muscle relaxant Baclofen is the most commonly studied medication in the literature. As a GABA agonist it inhibits the reflexes at the spine by increasing GABA level. This impedes the release of neurotransmitters at the synaptic level and reduces SRPE by inhibiting cavernous muscles contraction. Historically it was used for priapism associated with neurological lesions. It was tried on 17 patients with SRPE, using increasing doses from 10 to 75 mg depending on the development of side effects. Partial response was observed in the majority of patients. Complete remission was obtained in 37%; only one patient didn’t respond. Half of the patients relapsed after treatment discontinuation. Mild adverse reactions are usually reported in > 10% of the patients due to CNS depression: dizziness, sedation, nausea, headache and hypotension [26, 30].

## Conclusion

Based on the limited number of treatment trials and reported cases, the low level of evidence and the lack of randomized clinical trials, no treatment consensus for SRPE can be established. Treatment decision so far, is based on reports of treatment success, where remission was sustained, and the medication was tolerated by the patients with minimal side effects. From the data available in literature, Baclofen is the more commonly used medication. It has a tolerable profile of adverse effects. PED5 inhibitors are considered potential treatments and are also already widely used and tolerated for other indications, but till now, only 2 successful trials have been reported with SRPE. Cinitapride is very promising, but only one case was studied. No adverse effects were reported. Clozapine can be very dangerous although highly effective.

**Fig. 2:**
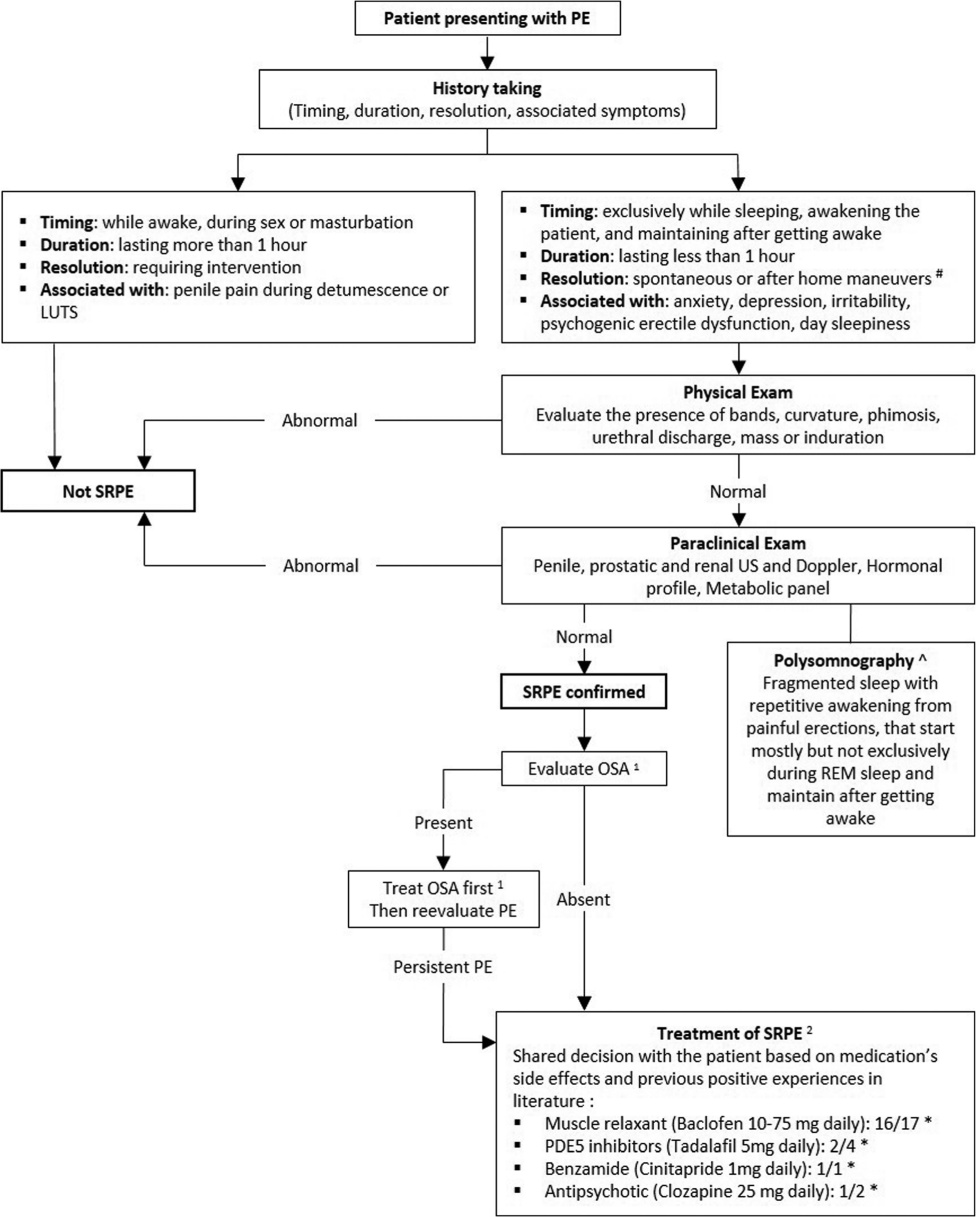
Proposed algorithm for the evaluation and management of sleep related painful erections. Abbreviations: PE: Painful erection; LUTS: Lower urinary tract symptoms; SRPE: Sleep related painful erection; US: Ultrasound; REM: Rapid eye movement; OSA: Obstructive sleep apnea; PDE: Phosphodiesterase. * Numerator: number of positive experiences; Denominator: total number of treated cases. # Home maneuvers include: walking, urinating, cold showers, cycling. ^ Pathognomonic, but not necessary for diagnosis [27, 30]

We propose at the end of our review a useful tool for clinicians (Fig. 2): an algorithm for the management of SRPE to facilitate their access to the literature without exhaustive return to cases reports and series upon each case faced.

Medications at the end of the algorithm are presented in a descending order of ratio of success of the well tolerated medications that were not abandoned.

## Data Availability

Not applicable.

## Abbreviations

cGMP: Cyclic guanosine monophosphate
CM: Cavernous muscles
CNS: Central nervous system
CPAP: Continuous positive airway pressure
GABA: Gamma aminobutyric acid
ICSD: International Classification of Sleep Disorders
MAOI: Monoamine oxidase inhibitors
NPT: Nocturnal penile tumescence
OSA: Obstructive sleep apnea
PDE5: Phosphodiesterase type 5
REM: Rapid eye movement
SRPE: Sleep related painful erection

## References

[1] Calvet U (1999). Painful nocturnal erection. Sleep Med Rev.

[2] Steiger A, Benkert O (1989). Examination and treatment of sleep-related painful erections-a case report. Arch Sex Behav.

[3] American Academy of Sleep Medicine (2001). International classification of sleep disorders, revised: Diagnostic and coding manual.

[4] Thorpy MJ (2012). Classification of sleep disorders. Neurotherapeutics..

[5] American Academy of Sleep Medicine (2014). International Classification of Sleep Disorders.

[6] Karsenty G, Werth E, Knapp PA, Curt A, Schurch B, Bassetti CL (2005). Sleep-related painful erections. Nat Clin Pract Urol.

[7] Vreugdenhil S, Weidenaar AC, de Jong IJ, van Driel MF (2017). Sleep-related painful erections—a case series of 24 patients regarding diagnostics and treatment options. Sex Med.

[8] Bai W-J, Hu H-B (2018). Considerations on priapism. Zhonghua Nan Ke Xue.

[9] Dai A-J, Li M, Wang L-L, Liu T, Wang X-H, Huang Y-H (2018). Improvement of erection related incision pain in circumcision patients using interrupted rapid eye movement sleep: a randomized controlled study. J Urol.

[10] Hu H, Cheng Y, Guan X, Li P, Chen Z, Dong B (2016). Diagnosis and management of sleep-related painful erections: a report of 9 cases. Zhonghua Nan Ke Xue.

[11] Menéndez López V, Mora Rufete A, Prieto Chaparro L, Galán Llopis JA, Fernández Puentes C, García López F (1999). Painful erections related to sleeping. Actas Urol Esp.

[12] Zhang J, Xiao Y, Li H (2019). Sleep-related painful erection in a patient with obstructive sleep apnea syndrome. Int J Impot Res.

[13] Kuhadiya ND, Desai A, Reisner M (2014). Sleep-related painful erections in an elderly man successfully treated using clonazepam. J Am Geriatr Soc.

[14] Szücs A, Janszky J, Barsi P, Erdei E, Clemens Z, Migléczi G (2002). Sleep-related painful erection is associated with neurovascular compression of basal forebrain. J Neurol.

[15] Ferini-Strambi L, Montorsi F, Zucconi M, Oldani A, Smirne S, Rigatti P (1996). Cardiac autonomic nervous activity in sleep-related painful erections. Sleep..

[16] Nair HKR (2018). Microcurrent as an adjunct therapy to accelerate chronic wound healing and reduce patient pain. J Wound Care.

[17] Lucky MA, McGuinness LA, Floyd MS, Azhar U, Shanks JH, Li C (2014). Epithelioid haemangioma: a rare cause of painful erections and sleep deprivation. Int Urol Nephrol.

[18] Hoeh MP, Levine LA (2014). Prevention of recurrent ischemic priapism with ketoconazole: evolution of a treatment protocol and patient outcomes. J Sex Med.

[19] Ukoli FA, Lynch BS, Adams-Campbell LL (2006). Radical prostatectomy and quality of life among African Americans. Ethn Dis.

[20] Hutter H-P, Moshammer H, Wallner P, Kundi M (2006). Subjective symptoms, sleeping problems, and cognitive performance in subjects living near mobile phone base stations. Occup Environ Med.

[21] Szúcs A (2004). Neurological aspects of some sleep disorders. Ideggyogy Sz.

[22] Lottman PE, Jongen PJ, Rosier PF, Meuleman EJ (1998). Sexual dysfunction in men with multiple sclerosis--a comprehensive pilot-study into etiology. Int J Impot Res.

[23] Schenck CH, Mahowald MW (1996). REM sleep parasomnias. Neurol Clin.

[24] Karacan I, Hursch CJ, Williams RL, Thornby JI (1972). Some characteristics of nocturnal penile tumescence in young adults. Arch Gen Psychiatry.

[25] Thorpy JM (1990). Handbook of Sleep Disorders.

[26] Barnhoorn PC, Gianotten WL, van Driel MF (2018). Sleep-related painful erections following sexual intercourse. Arch Sex Behav.

[27] Ferré A, Vila J, Jurado MJ, Arcalis N, Camps J, Cambrodi R, et al. Sleep-related painful erections associated with obstructive sleep apnea syndrome. Arch Sex Behav. 2012;41:1059–63. 10.1007/s10508-011-9894-2 22350120

[28] Ferini-Strambi L, Oldani A, Zucconi M, Castronovo V, Montorsi F, Rigatti P (1996). Sleep-related painful erections: clinical and polysomnographic features. J Sleep Res.

[29] Chiner E, Sancho-Chust JN, Llombart M, Camarasa A, Senent C, Mediero G (2010). Sleep-related painful erection in a 50-year-old man successfully treated with cinitapride. J Sex Med.

[30] Vreugdenhil S, Weidenaar AC, de Jong IJ, van Driel MF (2018). Sleep-related painful erections: a meta-analysis on the pathophysiology and risks and benefits of medical treatments. J Sex Med.

[31] Abouda M, Jomni T, Yangui F, Charfi MR, Arnulf I (2016). Sleep-related painful erections in a patient with obstructive sleep apnea syndrome. Arch Sex Behav.

[32] Peever J, Fuller PM. The biology of REM sleep. Curr Biol. 2017;27:R1237–48. 10.1016/j.cub.2017.10.026 29161567

[33] Schmidt MH (2018). Sleep-related erection neurophysiology: a journey of discovery. Sleep Med.

[34] Chen JY, Hockenberry MS, Lipshultz LI (2018). Objective assessments of Peyronie’s disease. Sex Med Rev.

[35] Salonia A, Eardley I, Giuliano F, Hatzichristou D, Moncada I, Vardi Y (2014). European Association of Urology guidelines on priapism. Eur Urol.

[36] Delavierre D, Rigaud J, Sibert L, Labat JJ (2010). Approche symptomatique des douleurs péniennes chroniques. Prog Urol.

[37] Imprialos KP, Stavropoulos K, Doumas M, Tziomalos K, Karagiannis A, Athyros VG (2018). Sexual dysfunction, cardiovascular risk and effects of pharmacotherapy. Curr Vasc Pharmacol.

[38] Matthews BJ, Crutchfield MB (1987). Painful nocturnal penile erections associated with rapid eye movement sleep. Sleep..

[39] Descazeaud A, de La Taille A, Giuliano F, Desgrandchamps F, Doridot G (2015). Effets négatifs sur la sexualité des traitements médicamenteux des symptômes du bas appareil urinaire liés à l’hypertrophie bénigne de la prostate. Prog Urol.

